# The Use of Graphite Micropowder in the Finish Turning of the Ti-6Al-4V Titanium Alloy Under Minimum Quantity Lubrication Conditions

**DOI:** 10.3390/ma17246121

**Published:** 2024-12-14

**Authors:** Joanna Lisowicz, Witold Habrat, Krzysztof Krupa, Grażyna Mrówka-Nowotnik, Paweł Szroeder, Magdalena Zawada-Michałowska, Jarosław Korpysa

**Affiliations:** 1 Department of Manufacturing Techniques and Automation, Faculty of Mechanical Engineering and Aeronautics, Rzeszow University of Technology, 12 Al. Powstancow Warszawy Street, 35-959 Rzeszow, Poland; witekhab@prz.edu.pl; 2 Department of Material Science, Faculty of Mechanical Engineering and Aeronautics, Rzeszow University of Technology, 12 Al. Powstancow Warszawy Street, 35-959 Rzeszow, Poland; krupa@prz.edu.pl (K.K.); mrowka@prz.edu.pl (G.M.-N.); 3 Instytut of Physics, Kazimierz Wielki University, Powstańców Wielkopolskich 2, 85-090 Bydgoszcz, Poland; psz@ukw.edu.pl; 4 Department of Production Engineering, Mechanical Engineering Faculty, Lublin University of Technology, Nadbystrzycka 36, 20-618 Lublin, Poland; m.michalowska@pollub.pl (M.Z.-M.); j.korpysa@pollub.pl (J.K.)

**Keywords:** minimum quantity lubrication (MQL), graphite micropowder, turning, Ti-6Al-4V, titanium alloy

## Abstract

The use of the minimum quantity lubrication (MQL) method during machining leads to the reduced consumption of cooling and lubricating liquids, thus contributing to sustainable machining. To improve the properties of liquids used under MQL conditions, they are enriched with various types of micro- and nanoparticles. The purpose of this study was to investigate the effect of the addition of graphite micropowder (GMP) on tool life, cutting force components, and selected surface roughness parameters during the finish turning of the Ti-6Al-4V titanium alloy under MQL conditions. The addition of 0.6 wt% of GMP to the base liquid in machining under MQL conditions leads to an extension of tool life by 7% and 96% compared to machining with a liquid without the addition of GMP and dry machining, respectively. Mathematical models of the cutting force components and surface roughness parameters were developed, taking into account the change in cutting speed and feed. It was found that the use of a liquid with the addition of GMP extends the range of cutting parameters for which the shape of chips obtained is acceptable in terms of work safety. The novelty of this study lies in the use of a cutting fluid composed of bis(2-ethylhexyl) adipate and diester, enriched with graphite micropowder, which has not been extensively investigated for machining titanium alloys under MQL conditions.

## 1. Introduction

During machining, great attention is paid to cutting parameters and cutting tools, their geometry, and the materials [1]. However, cooling and lubrication also play an important role during machining processes. The use of various methods for cooling and lubricating the cutting zone leads to a reduction in the coefficient of friction between the workpiece and the tool and to a reduction in the cutting temperature. This leads to a reduction in tool wear, an extension of tool life, and a reduction in the thermal damage of workpiece materials [2].

Flood cooling is the most common method used in industrial practice to supply a cooling and lubricating medium to the cutting zone [3]. Typically, this method assumes a flow rate of more than 100 L/h [4]. Better machining efficiency can be obtained by using high-pressure cooling (HPC) [5]. In this method, the coolant pressure is up to 20 MPa for a flow of approximately 24 L/min [4].

However, considering ecological aspects and the need to strive for sustainable machining, an alternative to flood and high-pressure cooling may be machining with minimum quantity lubrication (MQL) and minimum quantity cooling and lubrication (MQCL). Minimum quantity lubrication (MQL) is applied when the main objective of the use of an active medium is lubrication. When cooling and lubrication are required, an oil-based emulsifier concentrate mixed with water (in ratios ranging from 4:96 to 10:90) is used as an active medium, and the method is called minimum quantity cooling and lubrication (MQCL) [6]. In the MQL and MQCL methods, the standard coolant and lubricant flow rates range from 10 to 500 mL/h.

There are many articles in which the influence of different liquids used under MQL and MQCL conditions on selected parameters for the cutting process have been compared. In the research, vegetable oils (rapeseed oil [7], soybean oil [8], coconut oil [9], sunflower oil [10], palm oil [11], and olive oil [12]) and mineral oils [13], as well as water-based emulsions, [14,15] have been used. The main conclusions that can be drawn from previous studies are that machining under MQL and MQCL conditions, compared to dry machining, leads to a reduction in cutting force, surface roughness parameters, and tool wear and the prolongation of tool life, broadening the range of cutting parameters in which the chip shape is acceptable in terms of work safety.

The desire to improve the properties of cutting fluids has led to the development of a new class of cutting fluids, usually used under MQL conditions, called nanoliquids [16]. A nanoliquid is a mixture of a base liquid and particles, ranging in size from a few nanometers to a few micrometers, suspended in the base fluid. The addition of micro- and nanoparticles is intended to improve the cooling and lubricating properties of the liquids used [17,18]. Examples of nanoparticles used in scientific work include nanodiamond particles [19,20], aluminum trioxide (Al_2_O_3_) [21,22], molybdenum disulfide (MoS_2_) [23,24], silicon dioxide (SiO_2_) [25,26], titanium dioxide (TiO2) [27,28], copper nanoparticles [29,30], nanoboric acid [31], multi-wall carbon nanotubes (MWCNTs) [32,33], and graphite [34,35].

Maruda et al. [36] explored the impact of the size and concentration of copper nanoparticles on tool vibrations and surface topography during the turning of 316L stainless steel. The study highlighted that smaller nanoparticle sizes, particularly 22 nm, improved machining performance. Usluer et al. [37] conducted an experiment to evaluate sustainability, mainly related to carbon emissions and production costs, during the turning of S235JR structural steel under MQL conditions with nanofluids enriched with multi-walled carbon nanotubes (MWCNTs) and MoS_2_ nanoparticles. Turning under MQL conditions with MWCNT addition resulted in a significant reduction in costs and carbon emissions compared to dry machining and to machining with hybrid nanofluids (enriched with a mixture of MWCNTs and MoS_2_ nanoparticles). Ngo [38] demonstrated that the use of fluids enriched with MoS_2_ and Al_2_O_3_ during the turning of 90CrSi (60–62 HRC) steel under MQL and MQCL conditions led to increased machining performance and to a significant reduction in the total cutting force. Ngoc et al. [39] explored hybrid Al_2_O_3_/MoS_2_ nanofluids, showing that they provided a lower and more stable cutting force, cutting temperature, and surface roughness compared to single nanoparticle fluids in hard turning. Kara [40] found that the addition of nano-Al_2_O_3_ to the base fluid during the MQL turning of hot work tool steel led to a lower cutting temperature and surface roughness and reduced tool wear. Eltaggaz [41] found that adding Al_2_O_3_ to the base fluid during the milling of inconel 718 under MQL conditions significantly lowered the cutting force and improved chip morphology.

Bai et al. [42] identified that a 0.5% concentration of Al_2_O_3_ nanoparticles in cottonseed oil offered the best tribological properties and reduced energy consumption during the milling of 45 steel. James and Mazaheri [43] studied the influence of Al_2_O_3_ addition to cutting fluid in the high-speed machining of the 2219 aluminum alloy, finding that a 0.5% concentration of nanoparticles led to an optimal surface finish and reduced heat generation in the cutting zone.

The wide application but limited machinability of titanium alloys make them the subject of many studies. The increase in ecological and health awareness leads to the search for environmentally friendly and, at the same time, effective solutions that can replace the conventional machining of titanium alloys. An example of such a trend is the machining of titanium alloys under MQL conditions with the use of cooling and lubricating nanoliquids.

Gupta et al. [44] evaluated the machining efficiency of a grade-2 titanium alloy under MQL cutting conditions by employing nanofluids during the turning process. Three different types of nanoparticles were used to prepare nanoliquids: graphite, molybdenum disulfide (MoS_2_), and aluminum oxide (Al_2_O_3_). The base oil was vegetable oil, and the concentration of nanoparticles was 3% by mass. The following MQL parameters were used: air pressure of 0.5 MPa and a nanoliquid flow rate of 30 mL/h. The minimum values for the cutting force, tool wear, surface roughness, and cutting temperature were obtained for nanoliquids with the addition of graphite nanoparticles with an optimal set of parameters. The study also employed adaptive neuro-fuzzy inference system (ANFIS) and response surface methodology (RSM) for predictive modeling, with ANFIS outperforming RSM in accuracy. Optimization via composite desirability showed graphite nano-fluid as the best lubricant for minimizing cutting forces and enhancing surface finish.

Hegab et al. [45] studied the turning effectiveness of the Ti-6Al-4V titanium alloy with the use of a cutting fluid enriched with MWCNTs under MQL conditions. The average diameter of the MWCNTs ranged from 13 to 20 nm and the length from 10 to 30 μm. Nanoliquids were prepared at concentrations of 0.2 and 4% by weight. The base oil was ECOLUBRIC E200, a vegetable oil. The following parameters of the MQL system were selected: air pressure of 0.5 MPa and a liquid flow rate of 40 mL/h. The best surface quality was obtained for MQL nanoliquids with a concentration of 2 wt% with the optimal set of parameters. Tool wear was significantly reduced with the use of nanofluids. A smaller chip thickness was also observed after the use of nanoliquids with the addition of MWCNTs.

Gupta and Sood [46] studied the effect of three different nanofluids on the roughness of the machined surface in the turning process of a grade-2 titanium alloy. The average diameter of the nanoparticles used in the experiment was 40 nm, and the nanoparticles applied were graphite, molybdenum disulfide (MoS_2_), and aluminum oxide (Al_2_O_3_). Nanoliquids were prepared on the basis of vegetable oil at a concentration of 3% by weight. The nanoliquids were administered under MQL conditions, with a liquid flow rate of 30 mL/h and air pressure of 0.5 MPa. It was found that the values of the surface roughness parameters decreased with the change in nanoliquids from alumina-based nanoliquids to graphite-based nanoliquids.

Kishawy et al. [47] conducted research on vegetable oil-based nanofluids (ECOLUBRIC E200) when turning Ti-6Al-4V titanium alloys under MQL conditions (fluid flow rate of 40 mL/h and air pressure of 0.5 MPa). Al2O3 nanoparticles (average diameter 22 nm) at concentrations of 0.0, 2.0, and 4.0% by weight were used to prepare the nanoliquids. It was observed that machining with the use of MQL nanoliquids was characterized by better results in terms of tool wear, roughness of the machined surface, and cutting power.

Kim et al. [48] conducted a series of micro-shoulder milling experiments on the Ti-6Al-4V titanium alloy. An MQL nanoliquid with the addition of nanodiamond particles (ND), with an average size of 35 nm, and CO_2_ gas cooling were used for the study. The following MQL cooling parameters were selected: a liquid flow rate of 10 mL/h and air pressure of 0.15 MPa. The nanoliquids were prepared by adding nanodiamond particles to vegetable oil with weight concentrations of 0.1, 0.5, and 1.0 wt%. Both the MQL nanofluid and CO_2_ gas cooling were found to be effective in reducing the milling force, coefficient of friction, surface roughness, and the wear of the cutting tool.

Gaurav et al. [49] evaluated the machining performance in terms of cutting force, cutting tool wear, and surface roughness when turning the Ti-6Al-4V titanium alloy. The tests were carried out for five cutting environments, including dry turning, MQL with pure base liquids (jojoba oil and mineral oil LRT 30), and MQL with nanoliquids. The nanoliquids were prepared by adding molybdenum disulfide (MoS2, average diameter of 80–100 nm) to the base liquids at concentrations of 0.1, 0.5, and 0.9 wt%. The MQL parameters were as follows: a liquid flow rate of 60 mL/h and air pressure of 0.6 MPa. The results show that jojoba oil, in its pure state and with the addition of nanoparticles, is a good substitute for the MQL mineral rolling oil available on the market. It was found that MQL turning with jojoba oil + MoS2 (0.1%) reduced the cutting force, roughness of the machined surface, and wear of the tool edges in the range of 35–47%.

Maruda et al. [50] conducted an experiment focusing on the influence of four different sizes of copper nanoparticles on the tool wear mechanism and tool wear indicators during the turning of the Ti-6Al-4V titanium alloy under minimum quantity lubrication conditions. It was observed that the use of the smallest nanoparticles (22 nm) led to a significant reduction in tool wear. Moreover, the performance of the machining with the use of the smallest nanoparticles was better than that with larger sizes of nanoparticles and dry machining.

Edelbi et al. [51] studied the machinability of the Ti-3Al-2.5V alloy during face milling under dual-nozzle minimum quantity lubrication conditions with the use of a cutting fluid enriched with ZnO and compared the results with Al_2_O_3_ nanofluids. It was concluded that the use of ZnO nanofluids achieved a better surface quality and less tool wear.

In summary, in recent years, many researchers have focused on minimum quantity lubrication (MQL) as an eco-friendly alternative to conventional methods of cooling and lubrication during machining. The amount of fluid used in the MQL method is significantly reduced; however, it still provides effective lubrication and cooling, which leads to reduced tool wear, lower cutting forces, and improved surface quality. The development of nanofluids—base liquids enhanced with nanoparticles—has further improved the performance of MQL systems. Studies show that adding nanoparticles like Al_2_O_3_, MoS_2_, and graphite to MQL fluids enhances their lubricating and cooling properties, resulting in reduced tool wear, improved chip formation, and an enhanced surface finish.

The literature review highlights that machining titanium alloys is a significant challenge due to the wide range of applications for these materials in industry, as well as the difficulties encountered during their machining. Additionally, the need to consider sustainable development is paramount. A promising approach is to machine these alloys under MQL conditions. Since the amount of fluid used in MQL is minimal, selecting a fluid with optimal cooling and lubricating properties is crucial. In this work, the impact of a fluid mixture of bis(2-ethylhexyl) adipate and diester on cutting force components, surface roughness, chip formation, and the microstructure of the surface layer during the turning of the titanium alloy Ti-6Al-4V was evaluated. The fluid was also enriched with graphite micropowder, and its effect on the machining process was additionally investigated.

The novelty of this article lies in the investigation of a cutting fluid composed of a mixture of bis(2-ethylhexyl) adipate and diester, enriched with graphite micropowder, for machining the titanium alloy Ti-6Al-4V under minimum quantity lubrication (MQL) conditions. This study explores the influence of graphite micropowder on cutting force components, surface roughness, chip formation, and the microstructure of the surface layer during turning. The work provides valuable insights into the potential of MQL with enhanced fluids for improving the efficiency and sustainability of titanium alloy machining.

The aim of the study was to determine the influence of the addition of graphite micropowder to a liquid based on bis(2-ethylhexyl) adipate in the finish turning of Ti-6Al-4V under MQL cutting conditions. The results obtained were compared with those obtained during dry machining.

## 2. Materials and Methods

The workpiece material was a titanium alloy Ti-6Al-4V with a two-phase structure (α + β). It is one of the most commonly used grades in the aerospace and electrical industries. The chemical composition of the alloy is shown in Table 1 based on the inspection certificate provided by the manufacturer made in accordance with the EN 10204:04 standard [52]. The test samples were delivered in the form of rods with a diameter of 100 mm.

**Figure 1:**
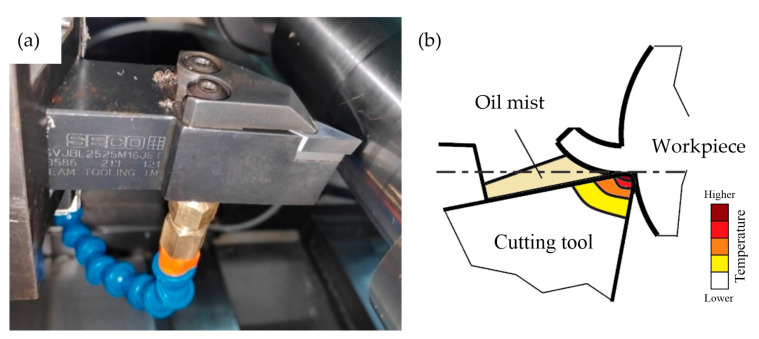
Internal cooling and lubrication liquid supply system: (**a**) tool holder, (**b**) oil mist delivery scheme.

The tests were carried out on a NEF 600 lathe. For the tests, a tool with a rhombic cutting insert VBGT160404-M3 made of uncoated HX-sintered carbide was used. A SVJBL2525M16 JET holder was used to clamp the cutting insert. It is able to supply a cooling and lubricating liquid mist to the rake face through specially prepared internal channels (Figure 1). A summary of the cutting parameters and the experimental setup used for the wear tests is shown in Table 2.

Two liquids (A3G0 and A3G60) made of bis(2-ethylhexyl) adipate and diester enriched with EP/AW additive were used for the MQL tests. Additionally, the A3G60 liquid was enriched with graphite micropowder (GMP) with a mass concentration of 0.60%.

The graphite micropowder C4N-98 (3000 mesh, carbon content 96%), known as GMP, was supplied by the Institute of Carbon Technologies Sp. z o.o. (Torun, Poland). Apart from drying, the material was used as received.

The microstructure of the graphite micropowder was analyzed by scanning electron microscopy (SEM, 1430 VP, LEO Electron Microscopy Ltd., Oberkochen, Germany) using a BSE detector. The surface elemental analyses were performed using an EDS Quantax 200 X-ray spectrometer equipped with an XFlash 4010 detector (Bruker Nano GmbH, Berlin, Germany).

**Figure 2:**
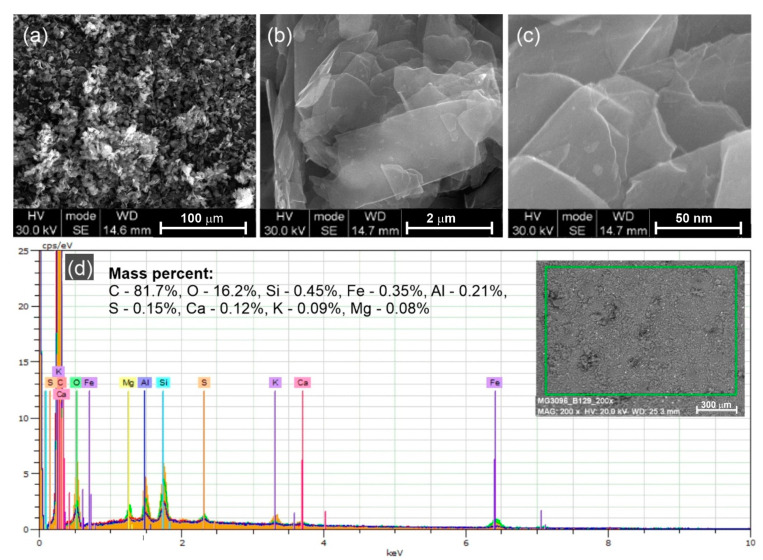
SEM images of GMP at different magnifications: (**a**) 1000×, (**b**) 50,000×, and (**c**) 200,000×; (**d**) EDX spectrum obtained from the area marked with a green rectangle with the results of the surface elemental analysis.

The morphology of GMP is shown in Figure 2a–c. In the low magnification SEM image (Figure 2a), a homogeneous distribution of grain size can be seen. The average size of the GMP was estimated as (6 ± 2) µm. The lamellar structure of the individual microplatelets becomes visible at higher magnifications (Figure 2b,c). It was noted that at an accelerating voltage of 30 kV, the graphite plates are partially transparent to the electron beam. Figure 2d shows the results of the surface elemental analysis carried out using the SEM-EDX technique. The high oxygen content (16 wt%) is associated with the oxidation of GMP on surface defects and microplatelet edges under ambient air. Trace amounts of elements such as Si, Fe, Al, S, Ca, K, and Mg were also observed.

The relationships between the cutting parameters of the process and the components of the cutting force, as well as the roughness parameters *Sa* and *Sz*, were modeled using the statistical response surface method (RSM), which is widely used in the modeling of cutting processes, including difficult-to-machine aerospace alloys. Due to the adopted range of the tested parameters (*f* = 0.1 ÷ 0.3 mm/rev and *v_c_* = 40 ÷ 120 m/min), a central composite face-centered (CCF) design was used. The experiment was designed using Design Expert 12 software, and the results, including the graphs and equations, were also generated using this software.

**Figure 3:**
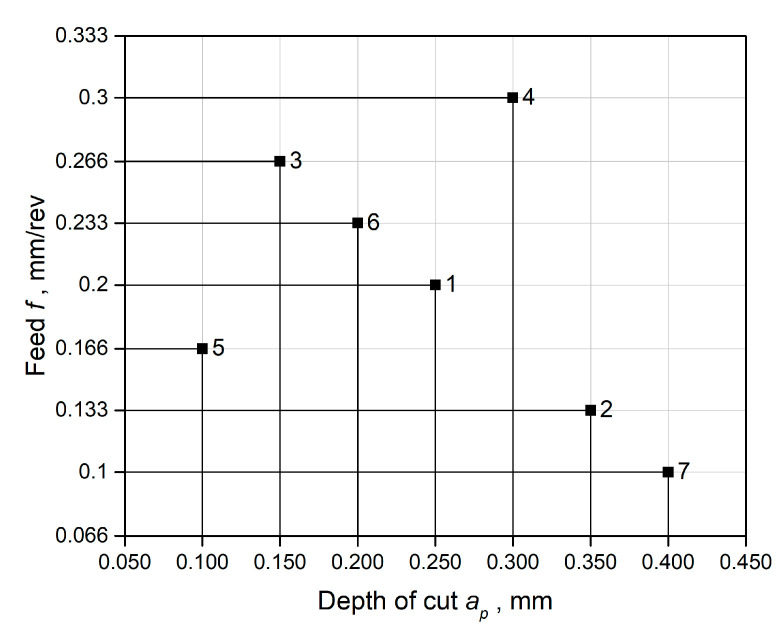
Location of research points in the field of chip-shaping control.

Due to the importance of chip geometry for the safety of the cutting process, tests were also carried out to control the shape of the chips after machining under MQL conditions with the A3G0 and A3G60 liquids. The research plan was based on the parameter space investigation (PSI) method, which allows the number of research points when planning the experiment to be minimized. The research points are arranged in multidimensional space so that their projections on the X and Y axes are evenly spaced relative to each other. Figure 3 shows the location of the research points in the field of chip-shaping control.

**Figure 4:**
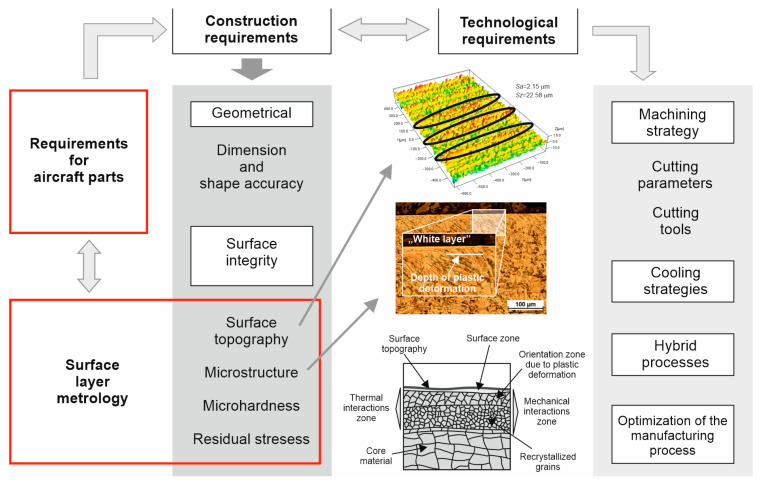
The structure and relationships occurring in the metrology of the surface layer of aircraft alloys after machining.

Titanium alloys are commonly utilized in the production of aircraft components. Due to the need for these parts to meet strict criteria, regarding not only geometry and dimensional precision but also the surface layer’s technological properties (Figure 4), control over the surface layer’s microstructural morphology was implemented. This layer refers to the external surface, which dictates the final shape of the machined part and the depth of the mechanical processing effects on the unmachined areas. The technological surface layer is distinguished by its unique topography and zones of thermo-mechanical interaction resulting from the cutting process.

## 3. Results

### 3.1. Tool Life

**Figure 5:**
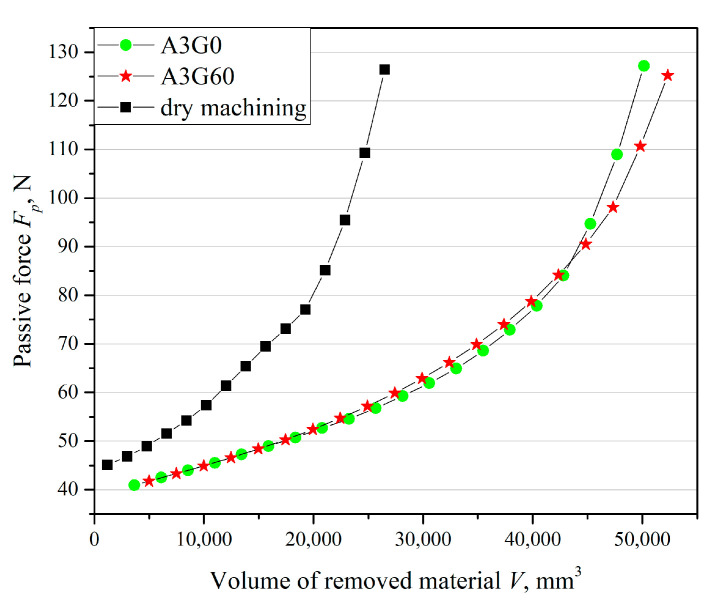
Passive force as a function of the volume of material removed.

Tool life was measured as the volume of material removed until an acceptable tool wear rate was reached. During previous studies, a close relationship was observed between the value of the resistive component of the cutting force *F_p_* and the tool wear index *VB_c_*. This observation made it possible to adopt a passive force value *F_p_* equal to 125 N as the tool wear criterion (Figure 5).

**Figure 6:**
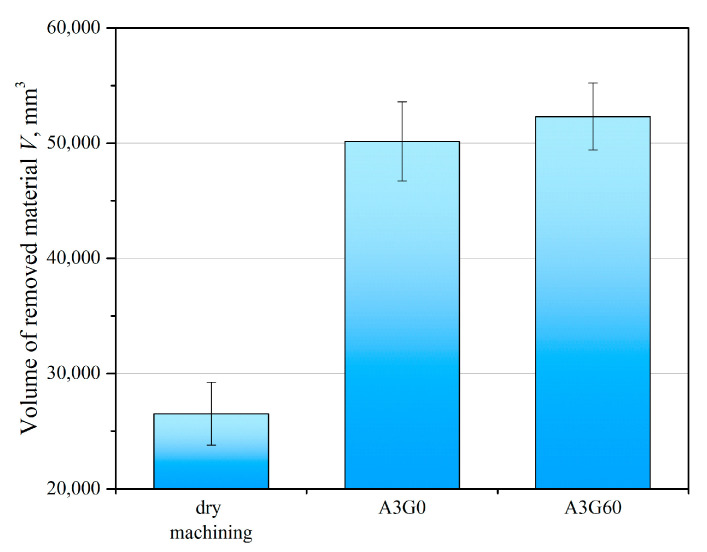
Volume of material removed until the tool wear criterion was reached for dry machining and machining under MQL conditions with the use of the A3G0 and A3G60 liquids.

It was shown that the addition of graphite micropowder at a concentration of 0.6 wt% to the base liquid during the finish turning of the Ti-6Al-4V alloy under MQL cutting conditions allowed an increase in the volume of removed material and thus extended tool life by 7% compared to machining with liquid without the addition of the micropowder. However, in relation to dry machining, the use of liquid with the addition of micrometric powder allowed tool life to be extended by 96% (Figure 6). The increase in tool life was due to the decrease in the value of the friction coefficient as a result of the use of GMP.

### 3.2. Modeling of the Cutting Force Components

**Figure 7:**
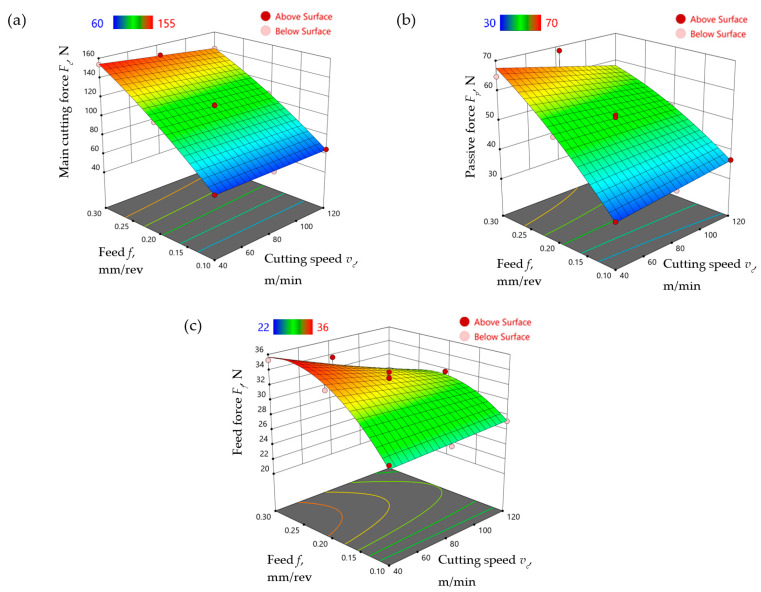
The influence of feed and cutting speed on the (**a**) main cutting force *F_c_*, (**b**) passive force *F_p_*, and (**c**) feed force *F_f_* in the finish turning of Ti-6Al-4V under MQL conditions with the use of the A3G0 liquid.

Models of the cutting force components were developed with the use of DesignExpert software, taking into account the change in feed and cutting speed for the finish turning of the Ti-6Al-4V titanium alloy under MQL conditions with the use of the A3G0 and A3G60 liquids. Equations (1)–(3) show the model relationships of the main cutting force *F_c_*, the thrust force *F_p_*, and the feed force *F_f_*, respectively, obtained for the A3G0 liquid. Figure 7 shows the response surface obtained according to Formulas (1)–(3).

(1) $$F_{c}=2.433+0.076v_{c}+671.955f-0.779v_{c}f-484.138f^{2}$$

(2) $$F_{p}=1.246+0.142v_{c}+308.314f-0.863v_{c}f-239.306f^{2}$$

(3) $$F_{f}=10.652+0.053v_{c}+189.847f-0.459v_{c}f-317.389f^{2}$$

**Figure 8:**
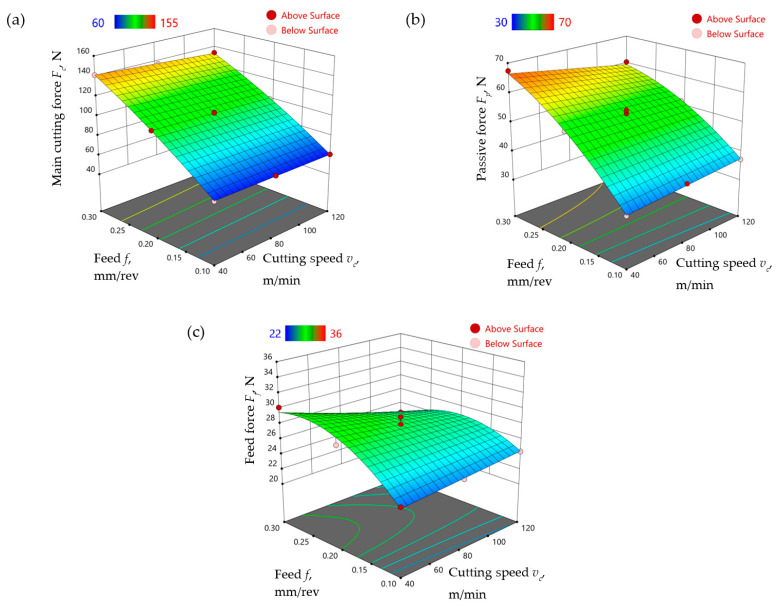
The influence of feed and cutting speed on the (**a**) main cutting force *F_c_*, (**b**) passive force *F_p_*, (**c**) feed force *F_f_* in the finish turning of Ti-6Al-4V in MQL conditions with the use of the A3G60 liquid.

Similarly, Equations (4)–(6) show the model relationships of the main cutting force *F_c_*, the thrust force *F_p_* and the feed force *F_f_*, respectively, obtained for the A3G60 liquid. Figure 8 shows the response surface obtained according to formulas (4)–(6).

(4) $$F_{c}=9.326+0.029v_{c}+573.006f-0.444v_{c}f-396.694f^{2}$$

(5) $$F_{p}=3.684+0.101v_{c}+326.240f-0.630v_{c}f-350.931f^{2}$$

(6) $$F_{f}=10.220+0.055v_{c}+150.071f-0.404v_{c}f-256.458f^{2}$$

Comparison of the models for both liquids, A3G0 and A3G60, led to the conclusion that the addition of graphite micropowder produced changes in tribological conditions and thus to changes in the values of the cutting force components.

**Figure 9:**
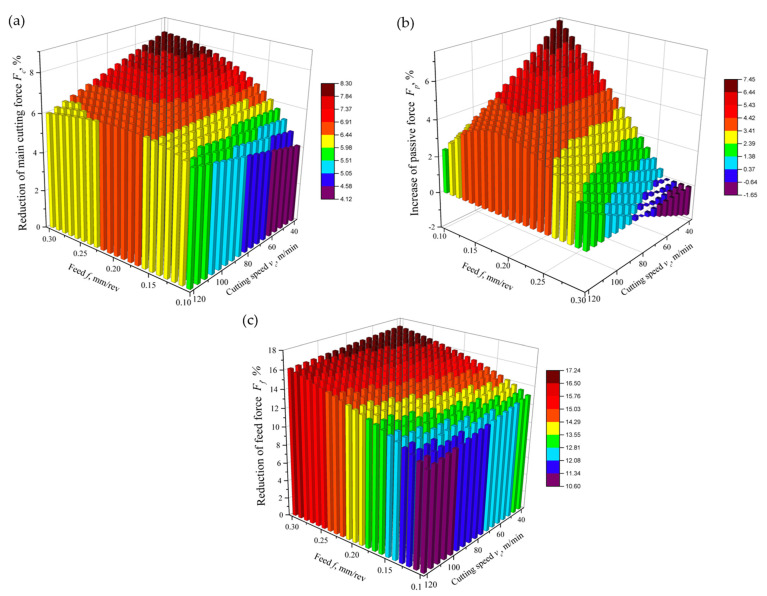
Changes in values of the (**a**) main cutting force *F_c_*, (**b**) passive force *F_p_*, and (**c**) feed force *F_f_* due to the addition of graphite micropowder to the base liquid in comparison to machining with a liquid without graphite micropowder.

In the case of the main cutting force *F_c_*, the addition of graphite micropowder led to a decrease in the force value of approximately 4–8%, with a greater difference noticeable for higher feed rates and lower cutting speed values (Figure 9a).

The greatest effect of the addition of GMP at a concentration of 0.6 wt% to the base liquid on the passive force *F_p_* was observed for the lower range of cutting parameters (*v_c_* = 40 m/min and *f* = 0.1 mm/rev). The passive force value *F_p_* increased by approximately 0.5–7% in the case of machining with the use of a liquid with the addition of graphite micropowder compared to machining with a liquid without graphite micropowder. A negligibly small reduction in passive force was observed only for feed values close to the upper limit of the tested range and cutting speed *v_c_* ≈ 40 m/min (Figure 9b).

The value of the feed force *F_f_* was reduced by about 11–17% during machining under MQL conditions with the use of a liquid with the addition of graphite micropowder in comparison to machining with a liquid without the addition of micropowder (Figure 9c).

### 3.3. Modeling of the Sa and Sz Roughness Parameters

**Figure 10:**
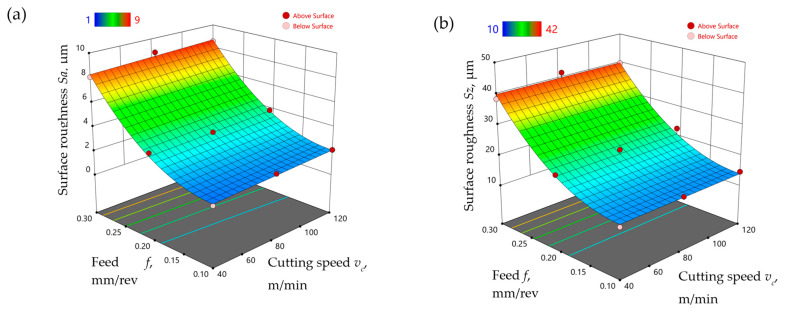
The influence of feed and cutting speed on the surface roughness parameters (**a**) *Sa* and (**b**) *Sz*, measured after the finish turning of Ti-6Al-4V under MQL conditions with the use of the A3G0 liquid.

Models of the surface roughness parameters *Sa* and *Sz* were developed, taking into account the change in feed and cutting speed for the finish turning of the Ti-6Al-4V titanium alloy under MQL conditions with the use of the A3G0 and A3G60 liquids. Equations (7) and (8) show the model relationships of the surface roughness parameters *Sa* and *Sz,* respectively, obtained for the A3G0 liquid. Figure 10 shows the response surface obtained on the basis of Formulas (7) and (8).

(7) $$Sa=3.006+0.005v_{c}-31.424f+161.172f^{2}$$

(8) $$Sz=19.940-123.437f+633.700f^{2}$$

**Figure 11:**
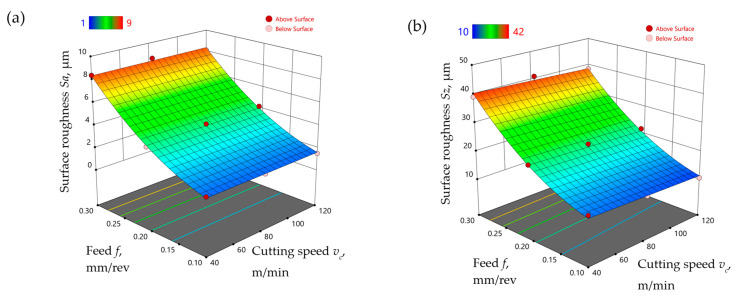
The influence of feed and cutting speed on the surface roughness parameters (**a**) *Sa* and (**b**) *Sz*, measured after the finish turning of Ti-6Al-4V under MQL conditions with the use of the A3G60 liquid.

Equations (9) and (10) show the model relationships of the surface roughness parameters *Sa* and *Sz*, respectively, obtained for the A3G60 liquid. Figure 11 shows the response surface obtained on the basis of formulas (9) and (10).

(9) $$Sa=1.152-5.230f+97.475f^{2}$$

(10) $$Sz=12.607-0.021v_{c}-29.752f+417.133f^{2}$$

**Figure 12:**
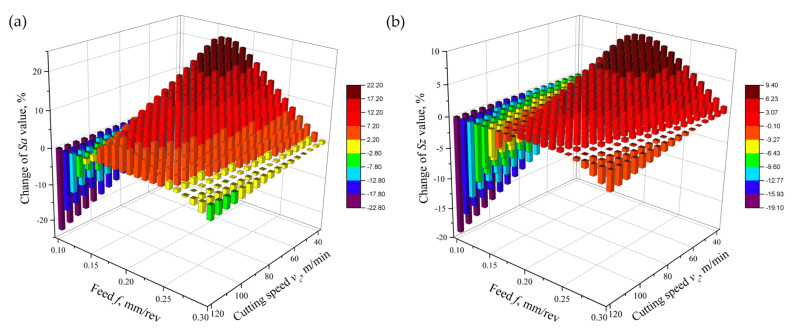
Changes in the values of surface roughness parameters (**a**) *Sa* and (**b**) *Sz* due to the addition of graphite micropowder to the base liquid in comparison to machining with a liquid without graphite micropowder.

It was observed that the addition of 0.6 wt% of GMP to the liquid used under MQL conditions during the finish turning of the Ti-6Al-4V alloy led to a decrease in the *Sa* and *Sz* parameters of the surface roughness after machining in the range of high cutting speeds and extreme feed values in the tested range. The greatest difference was observed for the speed *v_c_* = 120 m/min and the feed *f* = 0.1 mm/rev—there was a decrease in the values of the parameters *Sa* and *Sz* by about 23% and 19%, respectively, compared to machining with a liquid without the addition of GMP (Figure 12).

For a feed value in the range of about 0.15–0.25 mm/rev, an increase in the surface roughness parameters was observed, and these values increased with the decrease in cutting speed. For feed *f* ≈ 0.2 mm/rev and cutting speed *v_c_* ≈ 40 m/min, the values of *Sa* and *Sz* increased by approximately 22% and 9%, respectively, due to the use of a liquid with the addition of 0.6 wt% of GMP compared to treatment with a liquid without the addition of GMP.

**Figure 13:**
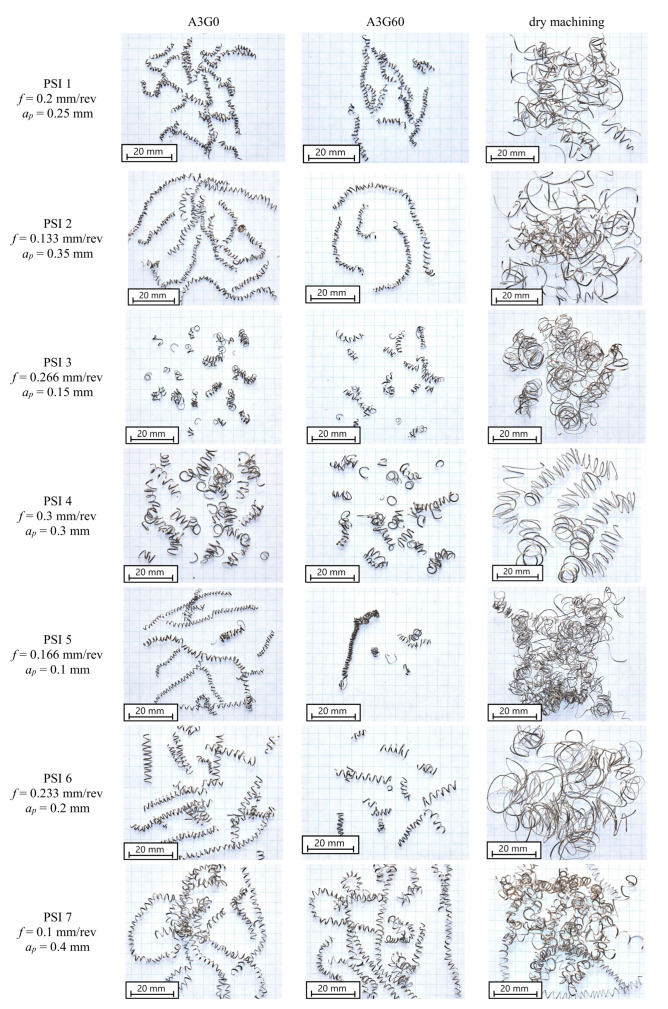
Chip shapes when finish turning Ti-6Al-4V.

Figure 13 illustrates the chip shapes of the Ti-6Al-4V titanium alloy produced during finish turning at PSI design test points under dry and MQL conditions using the A3G0 and A3G60 fluids. In MQL cutting with both liquids at ap = 0.15 mm and f = 0.266 mm/rev, as well as ap = 0.3 mm and f = 0.3 mm/rev, short open spiral chips were generated, which are considered safer for the working environment. Similar results were observed when finish turning with ap = 0.1 mm and f = 0.166 mm/rev and ap = 0.2 mm and f = 0.233 mm/rev using the A3G60 liquid under MQL conditions. However, tangled screw chips were observed with ap = 0.4 mm and f = 0.1 mm/rev under MQL conditions for both fluids. For other cutting parameters not mentioned here, long spiral chips were observed under MQL conditions. In contrast, tangled ribbon and spiral chips were generated under dry cutting conditions.

**Figure 14:**
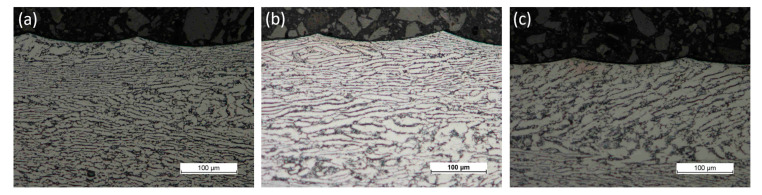
Examples of microstructures after (**a**) turning under MQL conditions without an additive, (**b**) turning under MQL conditions with the addition of 0.6 wt% of graphite micropowder, and (**c**) dry turning.

Figure 14 shows the microstructures of the surface layer of the Ti-6Al-4V alloy after finish turning under MQL conditions using a liquid based on bis(2-ethylhexyl) adipate with the addition of 0.6 wt% of graphite microplatelets (Figure 14a), without the addition of graphite powder (Figure 14b), and during dry turning (Figure 14c). No significant influence of thermo-mechanical interactions on plastic deformation in the surface layer with a visible deformation limit was observed for the tested range of process parameters. Also, no “white layer” was observed on the surface of the workpiece.

## 4. Discussion

In relation to the effect of adding graphite micropowder on tool life, an increase of 7% was observed compared to MQL machining without the addition of GMP and 96% compared to dry machining, which is a significant improvement. This may be due to the reduced friction coefficient resulting from the lubricating properties of graphite as an additive to the base fluid. There is also a potential effect of GMP on improved heat removal from the cutting zone. Comparing the obtained results with other studies on additives to MQL fluids in the machining of titanium alloys, such as the works of Gupta et al. [46] or Hegab et al. [47], the beneficial effect of lubricating particles was confirmed.

Analyzing the changes in the cutting force components, beneficial changes were observed after the addition of GMP. The reduction in the main force *F_c_* and the feed force *F_f_* by 4–8% and 11–17%, respectively, suggests improved cutting conditions. An increase in the *F_p_* resistance force by 0.5–7% may indicate changes in the chip formation mechanism. These interactions will affect the chip decohesion process and the quality of the machined surface. Analysis of tribological mechanisms allows for a better understanding of the effect of graphite microparticle addition on the cutting process of the Ti-6Al-4V alloy. Graphite, as a solid lubricant, creates a sliding layer on the contact surface of the tool and the machined material, which leads to reduced friction. The observed reduction in the main cutting force *F_c_* and the feed force *F_f_* when using the A3G60 fluid confirms the effectiveness of this mechanism. At the same time, the increase in the resistance force *F_p_* suggests that the graphite layer may slightly increase the tool pressure on the machined material. However, this effect is compensated by the overall reduction in friction, which translates into an extension of tool life compared to machining with a fluid without graphite addition.

The results regarding the roughness parameters *Sa* and *Sz* are ambiguous and depend on the cutting parameters. A decrease in roughness was observed at high cutting speeds and extreme feed values. Conversely, an increase in roughness occurred for average feed values, especially at low cutting speeds. These differences may result from the mechanisms of GMP action under different cutting conditions. The influence of graphite microparticle addition on surface roughness is dependent on the cutting parameters. The reduction in Sa and Sz parameters by 23% and 19%, respectively, at high cutting speeds and low feed rates may be attributed to improved heat dissipation from the cutting zone and stabilization of the chip formation process. Conversely, the increase in roughness observed at medium feed rates and lower cutting speeds may be caused by a change in the chip formation mechanism under these conditions.

The observed changes in chip shape using fluids with a GMP additive are important from the perspective of process safety. Short spiral chips are more beneficial. The chip formation mechanism changes as a result of using the GMP additive, which was observed both in the chip geometry and the previously mentioned changes in the range of the total cutting force components. Chip morphology analysis confirms that the addition of graphite influences the chip formation process, broadening the range of parameters for which favorable chip forms are obtained. These observations indicate the complexity of tribological interactions in the cutting process and highlight the need for further research on optimizing the composition of cutting fluids for different Ti-6Al-4V alloy machining conditions.

In terms of microstructure assessment, the deformation zone created as a result of plastic deformation and the thermal impact zone, where microstructural changes occur due to thermal processes (e.g., recrystallization and grain growth, phase transformations, chemical reactions) were examined. This creates metrological challenges in terms of surface layer measurements and requires broader studies covering a wider range of cutting parameters and different stages of wear.

The obtained results may have potential industrial applications due to the possibility of increasing the efficiency of machining titanium alloys. However, it is necessary to analyze the economic and ecological aspects of using MQL with the addition of GMP and the potential limitations and challenges related to the implementation of this technology. In particular, this concerns the challenges related to particle aggregation in the base fluid.

The addition of graphite micropowder to the MQL fluid shows promising potential for improving the finish turning process of the Ti-6Al-4V alloy, offering increased tool life, favorable changes in cutting forces, and potential benefits in terms of surface quality. Further studies are necessary to fully understand the mechanisms of action and optimize the process.

## 5. Conclusions

This study investigated the finish turning of the Ti-6Al-4V titanium alloy under MQL conditions using bis(2-ethylhexyl) adipate-based liquids, both without and with a 0.6 wt% addition of graphite micropowder (GMP). The following conclusions were drawn:

(1) The addition of 0.6 wt% of GMP increased tool life by 7% compared to machining with liquid without GMP and by 96% compared to dry machining due to reduced friction.
(2) Mathematical models showed that adding GMP decreased the main cutting force and feed force by 4–8% and 11–17%, respectively, but increased the passive force by up to 7%.
(3) GMP reduced surface roughness *Sa* and *Sz* by 23% and 19%, respectively, at higher cutting speeds and extreme feed values in the tested range. However, for feeds of 0.15–0.25 mm/rev, surface roughness increased as cutting speed decreased.
(4) The use of GMP extended the range of cutting parameters for which chip formation was safe and acceptable.

This research introduces a new cutting fluid formulation for the MQL machining of titanium alloys and provides insights into the benefits of GMP on machining performance.

Future studies could explore the optimization of GMP concentrations in cutting fluids for different machining conditions, as well as investigate its impact on other titanium alloys. Additionally, examining the long-term effects of GMP on tool wear and surface quality and comparing MQL with other cooling techniques could provide valuable insights for further improving machining performance.

## Figures and Tables

**Table 1:** The chemical composition of the workpiece material (expressed as the mass percentage of each constituent element).

Ti	C	Fe	N	Al	O	V	H	Y	Other
balance	0.0111	0.105	0.0065	6.41	0.176	4.17	0.0015	-	˂0.40

**Table 2:** Summary of the experimental setup and cutting parameters.

**Experimental Setup**	Workpiece material	Ti-6Al-4V
	Machine	NEF 600 lathe
	Cutting insert	VBGT160404-M3 HX
	Tool holder	SVJBL2525M16 JET
	Cutting fluid under MQL conditions	Fluids made of bis(2-ethylhexyl) adipate and diester: A3G0—0% of graphite micropowder (GMP) A3G60—0.6% of graphite micropowder GMP
**Cutting Parameters**	Fluid flow, mL/h	30
	Air pressure, MPa	0.7
	Depth of cut, mm	0.25
	Cutting speed, m/min	120
	Feed, mm/rev	0.1

## Data Availability

The original contributions presented in this study are included in the article. Further inquiries can be directed to the corresponding author.
